# Effects of Killari earthquake on the paleo-channel of Tirna River Basin from Central India using anisotropy of magnetic susceptibility

**DOI:** 10.1038/s41598-020-77542-9

**Published:** 2020-11-25

**Authors:** B. V. Lakshmi, K. Deenadayalan, Praveen B. Gawali, Saumitra Misra

**Affiliations:** 1 Indian Institute of Geomagnetism, New Panvel, Navi Mumbai, 410218 India; 2 Discipline of Geological Sciences, University of KwaZulu-Natal, Durban, 4000 South Africa

**Keywords:** Natural hazards, Solid Earth sciences

## Abstract

The Killari Earthquake (Moment magnitude 6.1) of September 30, 1993, occurred in the state of Maharashtra, India, has an epicenter (18°03′ N, 76°33′ E) located at ~ 40 km SSW of Killari Town. The ~ 125 km long basin of Tirna River, close to the Killari Town, currently occupies the area that has witnessed episodic intra-cratonic earthquakes, including the Killari Earthquake, during last 800 years. The anisotropy of magnetic susceptibility (AMS) study was performed on ~ 233 soft sedimentary core samples from six successions located in the upper to lower stream of the Tirna River basin in the present study in order to evaluate the effects of earthquake on the river flow dynamics and its future consequence. The AMS K_max_ orientations of the samples from the upper reach of the river section suggest that the sedimentation in this part of the river was controlled by a N–S to NNW–SSE fluvial regime with a low or medium flow velocity. In the middle reaches of the basin, an abrupt shift in the palaeo-flow direction occurred to W–E with low velocity flow. However, a NW–SE higher palaeo-flow regime is identified in the following central part of the basin in down-stream direction, followed by a low-velocity palaeo-flow regime at the lower reach of the Tirna basin. We attribute the sudden high flow velocity regime in the central part of the river basin to an enhanced gradient of the river that resulted from the reactivation of a NW–SE fault transecting the Tirna River basin at the Killari Town. As the NW–SE faulting in regional scale is attributed as the main cause of Killari Earthquake, the reactivation of this fault, thus, could enhance the further possibility of an earthquake in near future, and hence leading to devastating flood in the almost flat-lying downstream part of the Tirna River.

## Introduction

**Figure 1:**
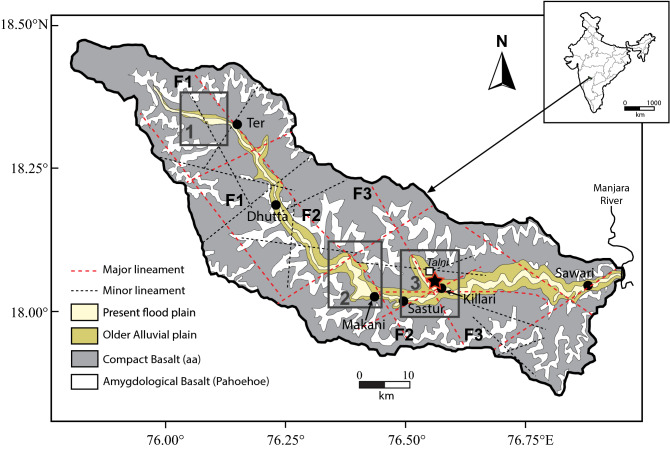
Geological map of the Tirna River basin showing the lineaments and location of studied outcrops (modified after Chetty and Rao^13^ and Babar et al^19^). The major (red dashed lines) and minor (black dashed lines) lineaments occurring along NE–SW, NW–SE, E–W and WNW–ESE directions. The sampling sites are: Ter, Dhutta, Makani, Sastur, Killari and Sawari. Red Star indicates epicenter of 1993 Killari Earthquake (M_w_ = 6.1). The dextral displacements of the Tirna River faults are shown in F1 and F2.

A devastating earthquake [Moment magnitude (M_w_): 6.1] occurred close to Killari town (18°03′ N, 76°33′ E) in Maharashtra state, India^1,2^ (Fig. 1), on September 30, 1993. This earthquake was considered as an example of intra-cratonic earthquake that occurred in a region of low background seismicity that resulted a poorly developed geomorphologic expression, and had long recurrence interval^2,3^. Multiple hypotheses were proposed in explaining the origin of this earthquake that include (a) rupture along a new fault plane^2–4^; (b) subduction of Indian plate beneath Tibetan plate^5–7^, and (c) flexural buckling of Indian plate to the north in contact with Tibetan plate^8^. The most recent hypothesis suggests that the Killari Earthquake resulted due to a deep crustal/lithospheric dynamics^9,10^. Some other opinion suggests that the existing faults were reactivated to generate the earthquake^11,12^. The Killari Earthquake produced a NW–SE trending deformation zone having a length of ~ 3 km and width of 300 m, which was associated with surface rupture^2,3^. The geomorphological features that resulted from such deformation includes relative subsidence and/or uplift, and small scale local upheavals^1,2^. A ~ 40 m long and 2–8 m wide, narrow elevated linear ridge is also observed close to the Killari Town^13^. Also, a 17 km long, NW–SE reverse fault that extends from Talni to Killari Town with a dip 45°SW was suggested to have produced from the focal mechanism of the main shock (Fig. 1)^5,14^, although an elaborate structural analyses of this fault is awaited. It was also suggested that the Killari Earthquake had resulted from the reactivation of pre-existing fault^5^.

**Figure 2:**
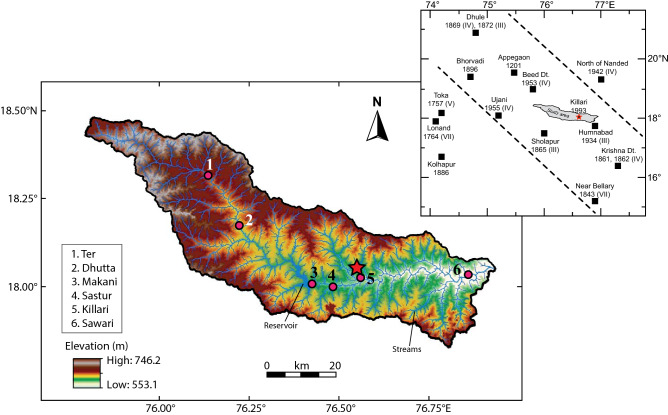
Digital elevation model (DEM) of Tirna River basin (Source: http://srtm.csi.cgiar.org) and inset map shows location of historic earthquakes around Killari (modified after Jain et al^2^ and Rajendran et al^5^).

The ~ 125 km long Tirna River, the subject of present study, is currently flowing towards the east along a channel that occupies ~ 10 km long WSW–ENE trending fault zone in the vicinity of the earthquake epicenter (Fig. 1). Several subsurface faults trending mainly NW–SE, and a few in N–S, NE–SW and ENE–WSW are also identified in satellite imageries crosscutting the Tirna River basin^13^. The relative movement along these NW–SE and ENE–WSW lineaments results in a well-developed mosaic of block structure^13^. However, the WSW–ENE lineament, along which the Tirna River is currently flowing close to the Killari Town, crosscuts all lineaments and exhibits sinistral strike-slip movement^13^. A re-investigation of the Tirna River basin has significance because this NW–SE trending river basin is confined in an area that contains epicenters of most of the earthquakes (M < 6.5) that occurred during the last ~ 800 years (Fig. 2 inset map).

The main focus of scientific research in and around Killari Town, till date, was mostly concentrated in evaluating the origin of 1993 earthquake^2–7,9,10,15^. However, no scientific study has been undertaken till date to evaluate the possible impact of earthquake on the regional environment in and around this area. The local agricultural economy and habitation in and around Killari Town mainly depends on the Tirna River^16^. Hence, in the present paper, we have attempted to identify the possible changes in palaeo-flow pattern of the Tirna River due to the earthquake using the technique of Anisotropy of Magnetic Susceptibility (AMS) of soft sediment cores collected from its floodplains, and tried to evaluate its future impact on the surrounding environment. An attempt has also been made to re-evaluate the future consequence of the Killari Earthquake.

## Geological setting

The ~ 125 km long Tirna River is an important tributary of the Manjara River that flows to the Godavari River basin in Maharashtra, India (Fig. 1). The Tirna Basin extends from 17°51′13′′ N to 18°28′33′′ N latitudes and 75°47′51′′ E to 76°57′14′′ E longitudes, and covers an area of ~ 8280 km^2^ with a maximum and minimum elevation of ~ 746 m to NW and ~ 553 m to E, respectively, with a general slope from ~ 10.3° towards NW to ~ 5.5° towards the east (Fig. 2). The main course of Tirna River is found to be controlled by a combination of NW–SE and WSW–ENE fault systems (Fig. 1). From its origin to the west upto the location Ter, the main course of the river changes from NW–SE to WSW–ENE; from Ter to Makani, the main channel of the river flows in NW–SE; finally, from Makani to Sawari, the main channel of the river orients WSW–ENE. The NE–SW lineament has poor control in shaping the direction of the river channel. The NW–SE fault, however, is found to be still active in controlling the course of the Tirna River, which are indicated by the dextral displacements of the Tirna River channel to the west of Ter along F_1_F_1_ fault (box 1 in Fig. 1) and along F_2_F_2_ fault close to Makani (box 2 in Fig. 1), and formation of a NW–SE tributary along F_3_F_3_ fault close to Killari (box 3 in Fig. 1). This basin has dendritic drainage pattern, where the lower reaches of the basin have streams of low gradient and more sinuosity. The river also shows the evidences of channel movement by avulsion largely controlled by the lineaments (Fig. 1). The older palaeo-levees exist in the form of ridges of around 4–5 m high at Ter, Killari, Sastur and Makani villages along the Tirna River flood-plain. The abnormally thick (~ 12–15 m) sediments are deposited near the Ter Village at the bed level of the Tirna River forming mounds^17,18^. In the exposure scale, these deposits are marked by curvilinear deposits over the silty or sandy bank deposits preserved along the older course of the river. The Quaternary deposits of the Tirna River basin have been divided into three informal lithostratigraphic Formations; viz., (i) the oldest dark grey silt Formation, (ii) the middle light grey silt Formation, and (iii) the youngest dark grayish brown silt Formation^19^. The early and late Formations of the Tirna River are thought to be stratigraphically equivalent to the Ramnagar and Bauras Formations, respectively, of the Narmada River alluvium^20,21^. The Quaternary sediments present in the area can be tentatively classified as pre-floodplain, floodplain, and pedi-plain deposits.

## Materials and methods

### Sampling and measurements

The sediment samples for the present study were collected in 8 cm^3^ cylindrical plastic containers (2.5 cm diameter and 2.2 cm height) using a portable soft sediment corer and were oriented using a magnetic compass. Two hundred and thirty three (233) oriented samples were collected from six sections along the Tirna River basin (Figs. 1, 2). The sampled sections were named as TR [Ter, number of sample (n) = 17], DT (Dhutta, n = 30), MK (Makani, n = 37), ST (Sastur, n = 26), KL (Killari, n = 57) and SW (Sawari, n = 66), which are located from the upstream to downstream of the Tirna River across the Killari Earthquake epicenter close to the Killari Town. Sampling process for this work focused primarily on fine‐grained sediments such as silt and clay samples and zones of pedogenically altered and disturbed horizons were avoided.

The laboratory analyses for rock magnetic investigations were carried out at the Indian Institute of Geomagnetism, Navi Mumbai, India. The measurement of low-field (300 Am^−1^ at 920 Hz) AMS for each specimen was carried out using a MFK1 kappabridge with measurements in 64 directions on three mutually orthogonal planes, using an automatic rotator sample holder. The azimuths and magnitudes of principal susceptibility axes (K_max_, K_int_, and K_min_) were calculated using SUFAR software supplied by AGICO, together with other magnetic anisotropy parameters such as anisotropy ratios, expressed as corrected degree of anisotropy (P′) and shape (T)^22^. The analysis of the AMS data was performed using the Anisoft 5 software.

Isothermal Remanent Magnetization (IRM) was imparted with a pulse magnetizer at a forward field of 20 and 1000 mT and at backward fields of 300 mT. All remanences were measured using a Molspin spinner magnetometer. The magnetization acquired at 1000 mT was defined as the saturation isothermal remanent magnetization (SIRM). The remanent coercive force (H_cr_) characteristic was obtained using a MMPM9 pulse magnetizer and a Molspin spinner magnetometer. Selected samples of each profile were subjected to high-temperature magnetization and hysteresis loop measurements in order to gain further insights into magnetic mineralogy and grain size. For representative samples, thermomagnetic runs of magnetic susceptibility (*k*–*T* curves) were performed using a Kappabridge KLY-4S (AGICO) equipped with a furnace. The sample was heated from room temperature to 700 °C and cooled back to room temperature in an argon atmosphere. Hysteresis loops and First-order reversal curves (FORCs) were obtained by an alternating gradient magnetometer Micromag 2900 with a maximum field of ± 1 T. Values of saturation magnetization (*M*_*s*_), saturation remanent magnetization (*M*_*rs*_), coercive force (*H*_*c*_) and coercivity of remanence (*H*_*cr*_) were calculated from the hysteresis loops. FORC diagrams were processed using the FORCinel software^23^.

### Anisotropy of magnetic susceptibility (AMS) method

The AMS of a rock sample corresponds to a symmetric, second-rank tensor that can be described by a triaxial ellipsoid with principal eigenvectors K_max_ > K_int_ > K_min_ representing the maximum, intermediate and minimum susceptibility axes respectively of the tensor ellipsoid^24^. The AMS technique has been widely used in geological science to evaluate the orientation of mineral fabric in igneous rocks or structurally deformed rocks^25–30^, naturally deposited soft-sediments^31–44^ and in laboratory deposited sediments^45–47^.

The experimental studies suggest that in fluvial environment, minimum susceptibility axes (K_min_) of the AMS ellipsoid always orient perpendicular (i.e., vertical) to the flow direction on a near horizontal depositional surface irrespective of weak to moderate (less than ~ 1 cm s^−1^) or strong (greater than or equal to ~ 1 cm s^−1^) current velocity^36,40,46–52^. The K_max_ axes, on the other hand, will be either parallel or perpendicular to the direction of flow when the current is either weak to moderate or strong, respectively^36,40,46–52^. Statistically, the long axis of K_min_ enveloping ellipse is parallel to the orientation of the main cluster of K_max_ axes in stereographic projection in low to moderate velocity environment and perpendicular in high velocity environment^36, 39,45–47,49^. The resultant shape of AMS ellipsoid of sediments is, therefore, prolate in fluvial environment, and the shape is oblate for sediments deposited in still water environment^47^. However, superimposition of some anisotropy on this oblate shape is possible if elongated grains roll on the shallowly sloping depositional surface and are preferentially aligned perpendicular to the slope^47^.

Following the pioneering laboratory experiment^45^, several authors^36–40,43,44^ applied these AMS techniques to study paleo-current directions in sedimentary rocks, especially in sediments of appropriate grain size. As the orientation of AMS ellipsoid in sedimentary environment is controlled by the direction and velocity of water flow^47^, this technique can be efficiently used to evaluate the palaeo-velocity of Tirna River because the main flow direction of this river is known to be dominantly eastward (Fig. 2).

Wide varieties of parameters have been used to describe the axial magnitude relationships of the susceptibility ellipsoid^53^. The corrected anisotropy degree expressed as P′ = exp [2{(ƞ_1_ − ƞ_m_)^2^ + (ƞ_2_ − ƞ_m_)^2^ + (ƞ_3_ − ƞ_m_)^2^}]^½^ where ƞ_1_ = ln K_1_, ƞ_2_ = ln K_2_, ƞ_3_ = ln K_3_, ƞ_m_ = (ƞ_1_ + ƞ_2_ + ƞ_3_)/3; and K_1_, K_2_ and K_3_ are maximum, intermediate and minimum susceptibility vectors respectively^22^. P′ shows the degree of anisotropy and reflects the eccentricity of the ellipsoid, i.e., the intensity of preferred orientation of minerals in a rock. The shape parameter, expressed as T = (2 ƞ_2_ − ƞ_1_ − ƞ_3_) / (ƞ_1_ − ƞ_3_), reflects the symmetry of the ellipsoid. It varies from − 1 (perfectly prolate shape) through 0 (triaxial shape, transition between prolate and oblate shape) to + 1 (perfectly oblate shape).

## Results

### Magnetic carriers

**Figure 3:**
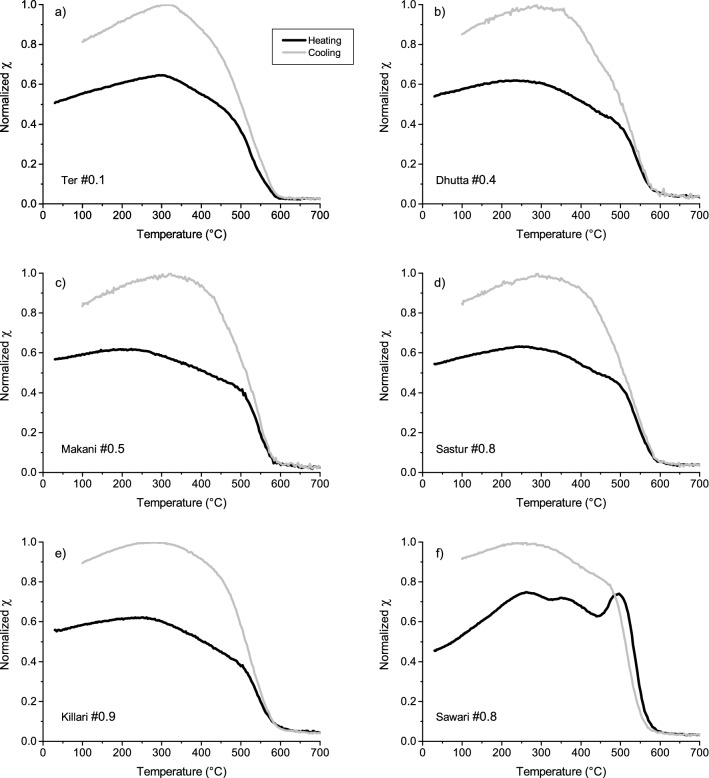
(**a**–**f**) Typical magnetic susceptibility versus temperature plots (χ-T curves) for representative samples from different depths from six studied sections Ter, Dhutta, Makani, Sastur, Killari and Sawari.

Temperature (T) dependent of magnetic susceptibility (χ) curves (χ–T) for selected samples from Ter, Dhutta, Makani, Sastur, Killari and Sawari sections are shown in Fig. 3. There is a decrease in χ values at ~ 300–400 °C and ~ 580 °C in heating curves (Fig. 3a–e) in almost all the measured samples. In sample Sawari 0.8, an increase in susceptibility is seen to be present with increase in temperature, which before dropping significantly at ∼ 350 °C. It is then seen to rise and is followed by a sharp drop at ∼ 580 °C (Fig. 3f). The sharp drops in susceptibility value at ∼ 350 °C and ∼ 580 °C indicate that the magnetic carrier in the samples is titanomagnetite and magnetite, respectively. The increase in χ between room temperature and the Curie temperature is typical for Ti-rich titanomagnetite^53,54^ for Ter, Dhutta, Makani, Sastur and Killari samples (Fig. 3a–e). For all the samples, susceptibility is seen to have recovered gradually with each successive step during cooling (Fig. 3), and the destruction of magnetic properties of Ti-rich titanomagnetite phases^53,54^ is the cause of the irreversibility of cooling curve.

**Figure 4:**
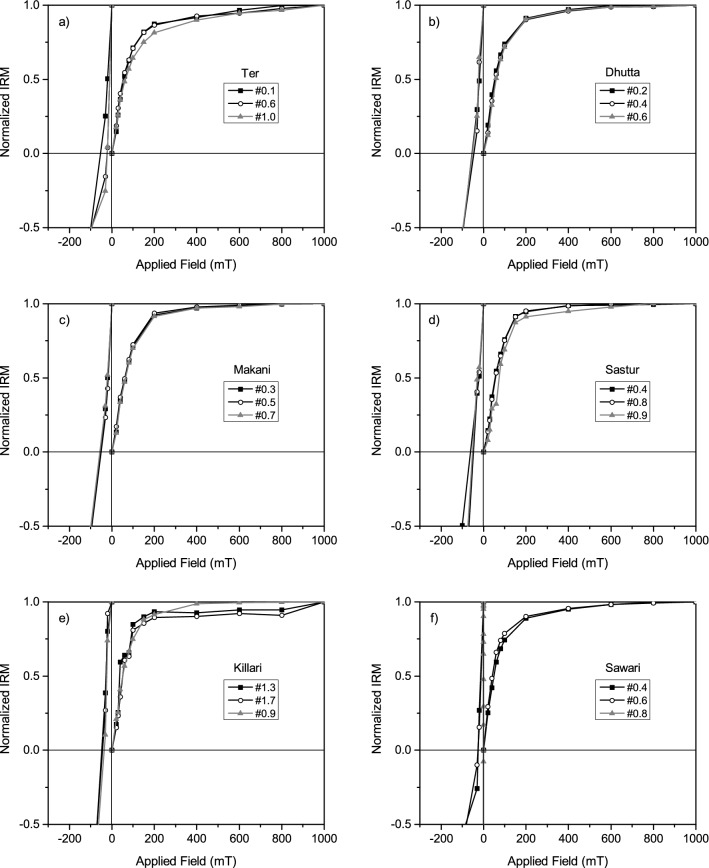
(**a**–**f**) Acquisition of isothermal remanent magnetization and backfield demagnetization for representative samples from the selected sites.

Figure 4 shows IRM acquisition curves for saturated isothermal remanent magnetization and remanent coercivity spectra for all the six sedimentary sections. The IRM acquisition curves for Ter, Dhutta, Makani, Sastur and Sawari samples are seen to increase rapidly from 0 to 150 mT, where ~ 70 to 80% of the maximum magnetization is acquired by 200 mT, and all samples achieve near complete saturation at 1000 mT (Fig. 4a–d,f). These observations suggest the low-coercivity ferrimagnetic mineral as the main magnetic carrier in our samples, with a possibility of having traces of high-coercivity magnetic mineral(s). For the Killari samples, < 95% of saturation is achieved at ~ 200 to 300 mT, indicating contribution is predominantly from a low-coercivity mineral (Fig. 4e). The values of remanent coercivity of representative samples (Fig. 4a–f) range between 25 and 80 mT, suggesting predominance of low-coercivity ferrimagnetic minerals.

### FORC diagrams and hysteresis parameters

**Figure 5:**
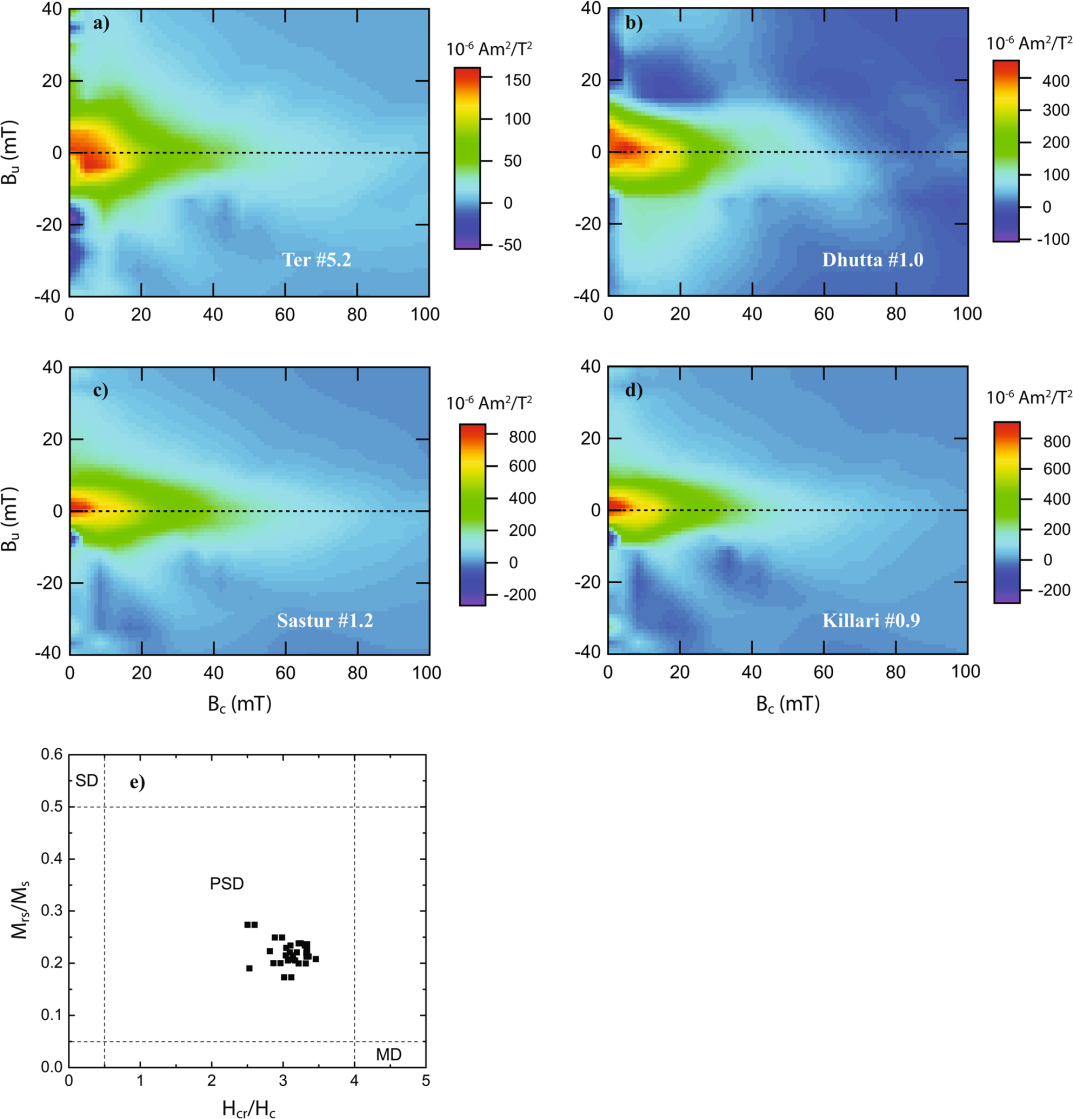
(**a**–**d**) Representative first-order reversal curve (FORC) diagrams for the representative samples from different depths, (FORCinel) (**e**) Results of Hysteresis measurements presented in a Day plot^64^.

To identify domain state that help in determining grain size and also to discriminate different components within a magnetic mineral assemblage^56–63^, FORC diagrams and hysteresis parameters are obtained for the samples (Fig. 5). The FORC diagrams of the samples show characteristics of vortex state magnetic grains by a peak at B_C_ ~ 10–15 mT with closed contours^57–61^. The hysteresis properties of the analyzed samples, viz., M_s_, M_rs_, H_c_ and H_cr_ are summarized in Fig. 5e. In M_rs_/M_s_ versus H_cr_/H_c_ plot^63^, the samples are seen to form a cluster within the pseudo-single domain (PSD) grain size. The hysteresis ratios are consistent with a dominant low-coercivity ferrimagnetic component magnetic grain size, most likely magnetite.

### AMS

**Figure 6:**
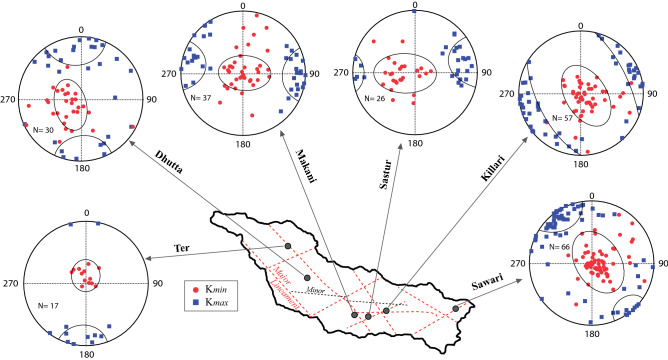
Equal area projections (lower hemisphere) of the directions of K_max_ (solid squares) and K_min_ axes (open circles) of AMS ellipsoids. The spreads in orientations of K_max_ and K_min_ axes are qualitatively indicated in the stereograms. The lineament pattern along the Tirna River basin also shown (dashed lines).

**Figure 7:**
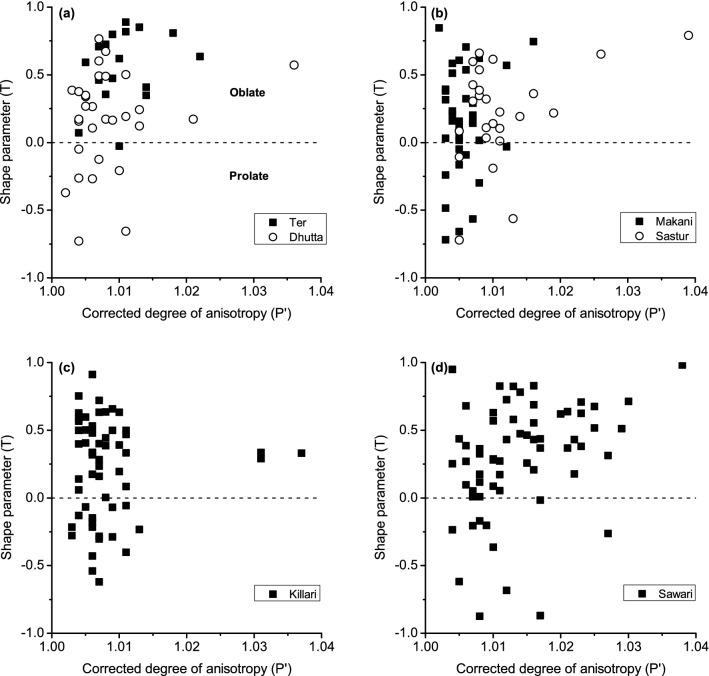
Variation of anisotropy degree (P′) and shape parameter (T) for the selected sites (**a**) Ter and Dhutta (**b**) Makani and Sastur (**c**) Killari and (**d**) Sawari.

Stereographic projections of K_max_ and K_min_ principal axes of AMS ellipsoids from all six sections are shown in Fig. 6. The K_min_ axes of Ter soft-sediment samples (N = 17) are mostly sub-vertical and dipping towards the north, where as the sub-horizontal K_max_ axes in this section are oriented primarily in N–S direction. In P′ versus T plots, Ter sedimentary samples plot only in the oblate quadrant with relatively low P′ values of < 1.015 (Fig. 7a).

Dhutta sedimentary succession, ~ 20 km SE of Ter, shows sub-vertical to inclined K_min_ axes mostly towards NNW-SSE, and horizontal to sub-horizontal K_max_ axes that have orientations mostly between NNW-SSE and NNE-SSW (Fig. 6). The long axis of K_min_ ellipse is statistically parallel or sub-parallel to the orientation of K_max_ axes. In P′ versus T plot, the majority of data from Dhutta succession plot in the oblate field (73%) with P′ (< 1.015), although subordinate spread in prolate field (27%) is also noticed (Fig. 7a). The majority of data from the Dhutta section are highly overlapping with those from the Ter succession in P′ versus T plot.

In the Makani section, in the middle reaches of the Tirna River, K_min_ axes are also vertical to incline that are oriented mostly towards E–W, although a few K_min_ axes show inclination towards NNE (Fig. 6). The corresponding K_max_ axes are horizontal to sub-horizontal and the majority of data are oriented in E–W. Like the Dhutta section, the long axis of K_min_ ellipse is also statistically parallel or sub-parallel to the orientation of K_max_ axes. In P′ versus T plot, data are plotted in oblate field in majority of cases (73%) with significant distribution in prolate quadrant (27%) as well. All the data have P′ < 1.015 (Fig. 7b).

In the Sastur succession, the majority of K_min_ axes are vertical to incline in E-W direction (Fig. 6), whereas the K_max_ axes are horizontal to sub-horizontal and are oriented in ENE–WSW direction in average. Like the Dhutta and Makani sections, the long axis of K_min_ ellipse is also statistically parallel or sub-parallel to the orientation of K_max_ axes. The P′ versus T plot reveals that the majority of data from Sastur succession lie in the oblate field (85%) with a few in prolate field (15%), wherein and the P′ values for the majority of data are ≤ 1.015 (Fig. 7b). However, a few data in the oblate field has P′ values between ~ 1.015 and 1.04.

In the Killari sedimentary section, K_min_ axes are vertical to incline in NW–SE (Fig. 6). The horizontal to sub-horizontal K_max_ axes are oriented from ESE–WNW to NE–SW. Unlike the previous three locations in upstream direction (Dhutta, Makani, Sastur), the trends of K_min_ axes in the Killari section are perpendicular to highly oblique to those of K_max_ axes. In P′ versus T plot, the Killari data are mostly distributed in the oblate field (72%), however, a spread in the prolate fields (28%) is also noticed. The P′ values are ≤ 1.015, however, a few samples have P′ values between 1.03 and 1.04. The Dhutta, Makani and Killari sections are distinct from the Ter section by having a low to important prolate component (~ 27%) in P′-T plot (Fig. 7a–c).

In the Sawari section, the distribution of K_min_ axes are sub-vertical to inclined, the inclined K_min_ axes are mostly oriented in NW–SE, although spread of a few sub-horizontal K_min_ axes towards NE is also seen (Fig. 6). The K_max_ axes are horizontal to sub-horizontal, and trend mostly in NW–SE direction. Unlike the Killari section, the long axis of the enveloping ellipse on the main cluster of K_min_ axes of the Sawari section is statistically parallel to the orientation of the majority of K_max_ axes, which is, however, similar to those of the Dhutta, Makani and Sastur sections. In P′ versus T plot, the Sawari data mostly plot in oblate field (85%) and a few in prolate fields (15%). The P′ values for these samples show higher range of variation of 1.03 (Fig. 7d).

## Discussion

The rock magnetic experiments on the present Tirna River soft sedimentary core samples help to infer ferrimagnetic minerals and PSD magnetic grain sizes predominantly (Figs. 3,4,5) in the samples. The step-like decrease in magnetic susceptibility at temperature 300–400 °C and 580 °C ranges suggests the mineral to be titanomagnetite with various Ti contents^54,55^ (Fig. 3a–f). The presence of magnetite/titanomagnetite is also supported by inference from the IRM and FORC study (Figs. 4 and 5). The decrease in magnetic susceptibility at 580 °C shows that magnetite grains were also present. Although, the river sediments, in general, contains more oxidized form of iron oxides, i.e. hematite, the presence of magnetite/titanomagnetite in the Tirna River sediments, therefore, suggests that the river sediments were subjected to protected surface alteration.

The experimental studies suggest that the orientation of K_max_ axes of AMS ellipsoids of river deposits is the function of two factors viz., direction and velocity of river flow^47^. As the flow direction of the Tirna River is constant mainly from NW to SE followed by W to E (Figs. 1 and 2), the orientations of K_max_ AMS axes can, therefore, be interpreted as the function of flow current of the river^41^. The orientations of K_max_ axes of AMS ellipsoid suggest that in the upper reaches of the Tirna River, at the Ter and Dhutta sections, the sedimentation was controlled mainly by the N–S and NNW–SSE flowing fluvial regimes, respectively (Fig. 6). The spread of main cluster of K_max_ axes in Dhutta section parallel (or sub-parallel) to the long-axis of K_min_ ellipsoid suggests medium to low palaeo-flow regime in this part of the Tirna River^37^. The low velocity of the palaeo-river flow in the upper reach of the Tirna River is also interpreted by mostly oblate shape of the AMS ellipsoids in P′ versus T diagrams^64–66^ (Fig. 7a). The sub-parallel relations of long-axes of K_min_ ellipsoids with the cluster distributions of the K_max_ axes in Makani and Sastur sections (Fig. 6) are also suggestive of low-velocity palaeo-fluvial environment^47,48^. However a subordinate spread in the prolate filed of Dhutta and Makani sections in the P′ versus T diagrams perhaps indicate local seasonal variation in river flow velocity.

In the central part of the river including the Makani, Sastur and Killari sections, the flow direction, as indicated by distribution of long-axes of K_max_ ellipsoids, seems to have shifted, in general, from E–W (in Makani and Sastur) to SW–NE (Killari) directions, although a wide variation of flow directions is observed in Killari section (Fig. 6). The near orthogonal relationship between the long-axis of K_min_ ellipsoid and respective clusters of K_max_ axes in the Killari section, suggesting high velocity with greater than ~ 1 cm s^−1^ in the central part of the Tirna River^36,47,48^. The high velocity of the palaeo-river flow in central part of the Tirna River is also suggested by mostly oblate to prolate shape of the AMS ellipsoids mainly from the Killari section in P′ versus T diagrams^29, 65–67^ (Fig. 7c). However, in the Sawari section the long-axis of K_min_ AMS ellipsoid is oriented parallel (or sub-parallel) to the distribution of the K_max_ AMS cluster (Fig. 6), indicating dominantly low-flow palaeo-regime in this section of the Tirna River^36^. The mostly oblate shape of the AMS ellipsoid in P′ versus T (Fig. 7d) also support low-velocity regime in this lowest reach of the Tirna River.

The WNW–ESE Tirna River basin presently occupies an area that has been active seismologically for the last ~ 800 years^2, 5^ (Fig. 2 inset map), and this seismicity is proposed to have been propagated by movement along a major NW–SE inter-cratonic reverse fault situated close to the Killari^3,5^, although a detail structural information on this fault is awaiting (Fig. 1). The moderate dextral displacements of the main Tirna River channel along the F_1_F_1_ fault to the west of Ter, and F_2_F_2_ fault to the west of Makani (Fig. 1) indicate that the NW–SE fault system is still active at present post-dating the formation of Tirna River. Therefore, it can be concluded that a possible reactivation along the NW–SE F_3_F_3_ fault, crosscutting the Tirna River channel to the west of Killari (Figs. 1, 6), in the recent geological past has enhanced the gradient of the Tirna River towards the east of this lineament. This recent movement along this fault plane, in turn, increases the slope of this part of the river channel resulting relatively high velocity of river flow in the central section of the Tirna River basin close to this fault zone at Killari during monsoon.

Hence, further movement along this presently active NW–SE fault could lead to the possibility of a major earthquake in this area in future. A fresh movement along this reverse fault could have further increased the gradient of the Tirna River close to the Killari town. This could lead to the possibility of sudden flash flooding in the Sawari section and to its downstream direction, to the east, particularly during the monsoon season. So no further habitation close to the Sawari section of the Tirna River is recommended on this study. However, if the movement along major intra-craton major fault system development in the Deccan Traps basalts was responsible for repeated earthquake in this area^2–7,11^, a detail regional analyses of the existing fault system as well as rock mechanical data are important for further prediction of earthquake in this area.

## Conclusions

In this present study, we determine paleo-flow directions of the Tirna River sediments using anisotropy of magnetic susceptibility with the help of rock magnetic studies. The AMS study results are summarized below:

- AMS analyses indicate that the recent sedimentation in the upper reaches of Tirna River (i.e., Ter and Dhutta sectors) was dominated by N–S to NNW–SSE fluvial regime with low to medium flow velocity (< 1 cm s^−1^).
- Low fluvial velocity (< 1 cm s^−1^) with an abrupt shift of flow direction to E–W was observed in the middle reaches of the river in Makani and Sastur sectors. In the lower reach of the Tirna River, in the Sawari section, low fluvial velocity is also observed in NNW–SSE direction.
- High flow velocity (> 1 cm s^−1^) with a SW–NE flow direction was observed in central section of Tirna River in the Killari sector. This sudden change to high fluvial velocity in the central section was resulted due to higher slope of the river valley in this area due to the NW–SE faulting transecting the river channel at Killari sector.
- As the Killari Earthquake is sensitive to regional intra-cratonic faulting^2–7,11^, an in depth study on the regional fault system along with a detail AMS study on Tirna River soft sedimentary cores are important for better understanding the possibility of further earthquake in future. This is because further movement along these fault system could have affects the flow pattern of Tirna River leading to the possibility of future flooding in this area.
